# Environmental enrichment is associated with enhanced novel object recognition performance and an increased proportion of smooth endoplasmic reticulum–containing spines in the dentate gyrus of *Septin3*^−/−^ mice

**DOI:** 10.1186/s13041-026-01315-0

**Published:** 2026-05-28

**Authors:** Natsumi Ageta-Ishihara, Naoki Fuse, Ayako Suzuki, Yugo Fukazawa, Makoto Kinoshita

**Affiliations:** 1 Department of Biomolecular Science, Faculty of Science, Toho University, 2-2-1 Miyama, Funabashi, Chiba, 274-8510 Japan; 2 Department of Molecular Biology, Division of Biological Sciences, Nagoya University Graduate School of Science, Nagoya, Japan; 3 Division of Brain Structure and Function, Faculty of Medical Science, University of Fukui, Fukui, Japan

**Keywords:** Septins, Septin-3, Environmental enrichment, Novel object recognition memory, Smooth endoplasmic reticulum, Hippocampus, Dentate gyrus

## Abstract

Septin-3 is a neuron-enriched member of the septin family, a group of GTP-binding cytoskeletal proteins. We previously reported that Septin-3 supports late-phase long-term potentiation (L-LTP)–induced entry of smooth endoplasmic reticulum (sER) into dentate gyrus (DG) spines, and that *Septin3*^−/−^ mice show reduced numbers of sER-containing DG spines and reduced performance in 24-h novel object recognition. Environmental enrichment (EE) enhances experience-dependent plasticity, raising the possibility that EE could modulate cognitive performance and spine sER phenotypes in *Septin3*^−/−^ mice. Here, we tested whether 4-week EE exposure enhances novel object recognition performance in *Septin3*^−/−^ mice and whether such behavioral changes are accompanied by changes in the proportion of sER-containing DG spines. Male *Septin3*^−/−^ mice were housed in standard housing (SH) or EE for 4 weeks and then subjected to novel object recognition. *Septin3*^−/−^ mice exposed to EE showed significantly better performance than SH-housed *Septin3*^−/−^ mice in the 24-h test session. Using serial section transmission electron microscopy (ssTEM) and three-dimensional (3D) reconstructions of the DG in *Septin3*^−/−^ mice immediately after the novel object recognition test following 4 weeks of SH or EE housing, we found that EE increased synapse density and elevated the proportion of spines containing sER, whereas spine volume and postsynaptic density (PSD) area were not detectably altered. Together, these results show that EE enhances 24-h novel object recognition performance and is associated with an increased proportion of sER-containing DG spines in *Septin3*^−/−^ mice following the novel object recognition test.

**Supplementary Information:**

The online version contains supplementary material available at 10.1186/s13041-026-01315-0.

Septin-3 is a neuron-enriched member of the septin family, a group of GTP-binding cytoskeletal proteins implicated in compartmentalizing membrane microdomains and organizing subcellular architecture in neurons [1–3]. Septin-3 has also been implicated in Alzheimer’s disease [4, 5]. We previously reported that Septin-3 supports L-LTP–induced entry of sER into DG spines, and that *Septin3*^−/−^ mice show reduced numbers of sER-containing spines in the DG middle molecular layer (DG-MML) together with impaired 24-h novel object recognition [6, 7].

EE is a widely used experimental paradigm that promotes experience-dependent plasticity and is often associated with enhanced hippocampal learning and memory [8, 9]. For example, 2–4-month-old mice housed in EE for 4 weeks showed a higher novel object exploration rate than non-enriched controls [10]. Whether EE exposure alters behavioral and ultrastructural measures in *Septin3*^−/−^ mice has not been tested. Here, we asked whether 4-week EE exposure enhances novel object recognition performance in *Septin3*^−/−^ mice relative to SH and whether such behavioral changes are accompanied by changes in the proportion of sER-containing spines in the DG-MML.

To evaluate the impact of experience on phenotypes in *Septin3*^−/−^ mice, 2–3-month-old male *Septin3*^−/−^ mice were housed for 4 weeks in SH or EE and then subjected to a 6-day novel object recognition schedule (Fig. 1a). The schedule included four consecutive days of daily handling and arena habituation (Days 1–4), a 15-min training session with two identical objects (Day 5), and a 5-min test session with one familiar and one novel object (Day 6). *Septin3*^−/−^ mice exposed to EE exhibited higher novel object recognition indices than SH-housed *Septin3*^−/−^ mice (Fig. 1b). Specifically, both the preference index (50% indicating no preference between the novel and familiar objects) and the discrimination index (DI; 0 indicating no novelty bias) were increased in the EE group. Consistent with a shift toward novelty-directed exploration, the DI in EE-exposed *Septin3*^−/−^ mice was significantly greater than the chance level of 0 (one-sample *t* test, *p* = 0.0045), whereas the DI in SH-housed *Septin3*^−/−^ mice did not differ from 0 (*p* = 0.51). Thus, EE was associated with higher 24-h novel object recognition performance in *Septin3*^−/−^ mice relative to SH. To assess whether the EE-associated increase in novelty-directed exploration could be attributed to altered locomotor activity during the test session, we quantified the total distance traveled during the 5-min Day 6 test. Total distance did not differ between SH- and EE-housed *Septin3*^−/−^ mice (Fig. 1c), indicating that the group difference in novel object recognition indices was not accompanied by a detectable change in overall locomotion during the test. To further examine potential time-dependent differences within the test session, we analyzed distance traveled in 1-min bins across the 5-min test; no differences in minute-binned locomotion between housing conditions were detected (Fig. S1).

**Fig. 1:**
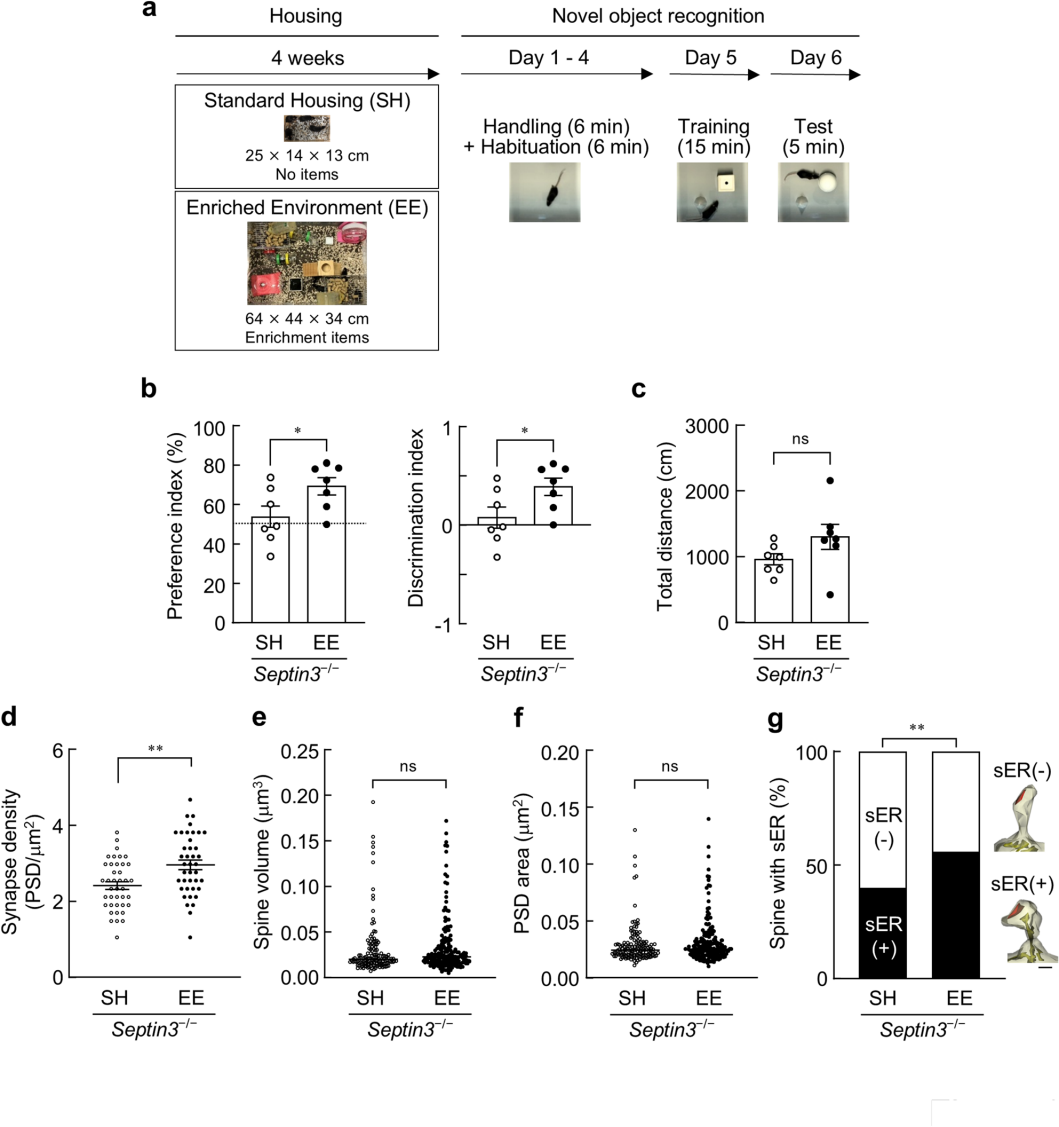
Environmental enrichment is associated with enhanced novel object recognition performance and an increased proportion of sER-containing DG spines after the novel object recognition test relative to standard housing in *Septin3*^*−/−*^ mice. **a–c**, Novel object recognition. **a**, Experimental design. Male *Septin3*^−/−^ mice (2–3-month-old at the start) were maintained in standard housing (SH) or an enriched environment (EE) for 4 weeks and then subjected to novel object recognition. Days 1–4, daily handling (6 min) plus habituation in the arena (6 min); Day 5, training (15 min); Day 6, test (5 min). **b**, Novel object recognition performance in the test session (Day 6; 1 day after training). Left, preference index, calculated as 100 × [time exploring the novel object] / ([time exploring the novel + familiar objects]); the dashed line indicates chance level (50%). Right, discrimination index, calculated as ([exploration time of the novel object] − [exploration time of the familiar object]) / ([exploration time of the novel object] + [exploration time of the familiar object]); 0 indicates no preference (chance level). *n* = 7 male *Septin3*^−/−^ mice per housing condition; two-tailed unpaired *t* test. Data are shown as mean ± SEM. *p < 0.05. **c** , Locomotor activity in the test session (Day 6), quantified as total distance traveled during the 5-min test. *n* = 7 male *Septin3*^−/−^ mice per housing condition; two-tailed unpaired *t* test. Data are shown as mean ± SEM. ns, not significant. **d–g**, Serial section transmission electron microscopy. **d**, Synapse density. *n* = 40 dissector pairs of sections taken from four areas across both hemispheres in the DG-MML in two 3–4-month-old male *Septin3*^−/−^ mice in each housing condition (SH and EE) for 4 weeks; two-tailed unpaired *t* test. Data are shown as mean ± SEM. **e**, Spine volume. *n* = 143 (SH) and 172 (EE) spines from *Septin3*^−/−^ mice after 4 weeks of housing; Mann-Whitney test. Data are shown as median. **f**, PSD area. *n* = 143 (SH) and 172 (EE) spines from *Septin3*^−/−^ mice after 4 weeks of housing; Mann-Whitney test. Data are shown as median. **g**, Percentage of spines containing sER. *n* = 143 (SH) and 172 (EE) spines from *Septin3*^−/−^ mice after 4 weeks of housing; Fisher’s exact test. Representative 3D reconstructions of dendritic spines (yellow) with or without sER (green), and PSD area (red), in the DG-MML. Scale bar, 100 nm. **p < 0.01, ns, not significant

Given our previous finding that *Septin3*^−/−^ mice exhibit impaired 24-h novel object recognition together with reduced numbers of sER-containing spines in the DG-MML [7], we next asked whether EE exposure was accompanied by ultrastructural changes in the DG-MML after the novel object recognition test. To this end, we performed ssTEM in the DG-MML immediately after the Day 6 test session following 4 weeks of SH or EE housing and quantified synaptic and spine parameters (Fig. 1d–g). Synapse density in the DG-MML was higher in EE-housed *Septin3*^−/−^ mice than in SH-housed *Septin3*^−/−^ mice (Fig. 1d), indicating an increase in synapse number under EE. We next quantified spine volume and PSD area. In contrast to synapse density, neither spine volume nor PSD area differed detectably between housing conditions (Fig. 1e, f). Thus, within the sensitivity of the present measurements, EE did not produce an overt shift in these size-related spine parameters in *Septin3*^−/−^ mice. Finally, we examined whether EE affects the prevalence of sER-containing spines. EE increased the proportion of spines containing sER in the DG-MML (Fig. 1g). Together, these ssTEM analyses indicate that, in *Septin3*^−/−^ mice, EE is associated with increased synapse density and a higher proportion of sER-containing DG spines, without detectable changes in spine volume or PSD area.

EE is widely used to engage experience-dependent plasticity [8, 9], yet how EE influences spine organelle states in defined genetic backgrounds remains unclear. In our previous study, *Septin3*^−/−^ mice exhibited both impaired 24-h novel object recognition and reduced numbers of sER-containing spines in the DG-MML [7]. On this basis, the present study examined whether EE exposure in *Septin3*^−/−^ mice was associated with changes in novel object recognition performance together with DG-MML ultrastructural measures. Here, in *Septin3*^−/−^ mice, 4-week EE exposure was associated with higher 24-h novel object recognition performance and a higher proportion of sER–containing spines in the DG-MML when assessed immediately after the novel object recognition test. EE also increased synapse density, whereas spine volume and postsynaptic density area were not detectably altered, suggesting a selective shift in synapse number and spine sER prevalence rather than global enlargement of postsynaptic structures. Notably, although Septin-3 regulates L-LTP–dependent sER extension into DG spines [6, 7], the present findings suggest that experience can modulate the prevalence of sER-containing spines even in its absence, potentially via compensatory or parallel pathways.

A limitation of the present study is the absence of a wild-type (WT) cohort under the same EE protocol. Therefore, although EE was associated with enhanced 24-h novel object recognition performance and altered DG-MML ultrastructure in *Septin3*^−/−^ mice, we cannot determine from the present dataset whether these effects are specific to the *Septin3*-deficient condition or reflect a more general response to EE. Future studies including WT mice housed under the same SH and EE conditions will be necessary to determine whether the present EE protocol exerts similar effects on object recognition memory and DG-MML ultrastructural measures in WT mice. Additional limitations include the use of male-only cohorts, focused ultrastructural sampling, and the fact that tissues were collected immediately after behavioral testing. Therefore, the present study does not distinguish whether the observed increase in synapse density and spine sER prevalence reflects baseline effects of prolonged EE exposure already present before behavioral testing, changes induced during the Day 5 training session, changes associated with the Day 6 test session, or a combination of these factors. Future work should define the mechanisms linking EE to spine sER remodeling and recognition memory.

## Methods

Methods are described in the Supplementary Materials.

## Supplementary Information

Below is the link to the electronic supplementary material.

Supplementary Material 1

## Data Availability

The datasets generated and analyzed during the current study are available from the corresponding author on reasonable request.

## Abbreviations

L-LTP: Late-phase long-term potentiation
sER: Smooth endoplasmic reticulum
DG: Dentate gyrus
EE: Environmental enrichment
SH: Standard housing
ssTEM: Serial section transmission electron microscopy
3D: Three-dimensional
PSD: Postsynaptic density
DG-MML: DG middle molecular layer
DI: Discrimination index
WT: Wild-type
