# Supplementary material for: Environmental enrichment is associated with enhanced novel object recognition performance and an increased proportion of smooth endoplasmic reticulum–containing spines in the dentate gyrus of Septin3−/− mice
Source: Mol Brain. 2026 May 28;19:42. doi: 10.1186/s13041-026-01315-0 (PMC13217727; doi:10.1186/s13041-026-01315-0)
Supplement: Supplementary file 1 — Supplementary Material 1 [file 13041_2026_1315_MOESM1_ESM.pdf]

Supplementary Materials

Methods

Animals

All animal experiments were reviewed and approved by the Institutional Animal Care and Use Committee of Nagoya University and were carried out in accordance with institutional guidelines. Mice were housed under a 12-h light/dark cycle with ad libitum access to food and water.

Septin3 knockout (*Septin3*$^{-/-}$) mice were generated from a previously established mutant line [1]. To minimize potential confounding effects of genetic background, the line was backcrossed for >10 generations onto C57BL/6J using a speed congenic strategy [2]. *Septin3*$^{-/-}$ mice were obtained by intercrossing *Septin3*$^{+/-}$ breeders. Only males were used. The age at each experiment is indicated in the figure legends.

Environmental enrichment exposure

Male *Septin3*$^{-/-}$ mice were assigned to either standard housing (SH) or an enriched environment (EE) for 4 weeks. The EE group was housed in a large cage (64 × 44 × 34 cm; L × W × H; 8 mice per cage) containing multiple enrichment items, including running wheels, tunnels, blocks, and a slide, using a modified version of the procedures described previously [3]. To maintain novelty, enrichment items and/or their spatial arrangement were replaced weekly at the time of routine cage exchange. The SH group was housed in standard cages (25 × 14 × 13 cm; 4 mice per cage), and cage exchange was performed on the same schedule as for the EE group.

Novel object recognition

Novel object recognition was performed following established procedures [2, 4]. Briefly, the task was conducted in a rectangular acrylic arena (250 × 290 × 290 mm) with one wall covered by white tape and the remaining walls made of gray acrylic. White ceramic objects were used (cube, 6 × 6 × 6 cm; sphere, 6 cm in diameter and 7.3 cm in height; octagonal pyramid, 4.6 cm in diameter and 7.4 cm in height). Object identity and location were counterbalanced across mice to minimize potential bias due to innate object preference or spatial preference.

Mice were habituated to the experimenter and the arena over four consecutive days (Days 1–4). Each day, mice were handled by the experimenter for 6 min and then were allowed to freely explore the empty arena for 6 min. On the training day (Day 5), two identical objects were placed near opposite corners along the diagonal of the arena, and the mouse was allowed to explore for 15 min. Memory was assessed 24 h later (Day 6) by replacing one familiar object with a novel object and allowing exploration for 5 min.

Behavior was recorded from above (10 fps). Videos were analyzed in ImageJ (NIH). Exploration was defined as frames in which the mouse oriented toward the object with the nose positioned within 2 cm of the object [5]. Episodes not considered exploratory, including climbing onto the object or immobility despite meeting the distance criterion, were excluded from scoring. The preference index (%) was calculated as 100 × [exploration time of the novel object] /([exploration time of the novel object] + [exploration time of the familiar object]), and the discrimination index was calculated as ([exploration time of the novel object] − [exploration time of the familiar object]) / ([exploration time of the novel object] + [exploration time of the familiar object]). Locomotion during the Day 6 test session was quantified as the total distance traveled over 5 min and as distance traveled in 1-min bins immediately after placement in the arena on Day 6.

Serial section transmission electron microscopy

Serial section transmission electron microscopy was performed following established procedures [2, 6, 7]. Briefly, ultrathin sections (50 nm) were prepared from the middle one-third of the dentate gyrus middle molecular layer (DG-MML) in male *Septin3*$^{-/-}$ mice immediately after the Day 6 test session following 4 weeks of SH or EE. Sections were imaged using a transmission electron microscope (JEM-1010; JEOL). Serial micrographs were aligned and three-dimensional reconstructions were generated using Reconstruct (SynapseWeb).

For morphometric analyses, spine volume, postsynaptic density area, and the presence of smooth endoplasmic reticulum were assessed by tracing all spines within predefined sampling fields across 30–35 serial sections acquired at 25,000× magnification. Synapse density was estimated using the physical dissector method from 30–35 serial sections acquired at 12,000× magnification. For each animal, four sampling fields in DG-MML were analyzed across both hemispheres. Analyses were performed blind to housing condition. After confirming no appreciable inter-animal differences within condition, measurements were pooled for statistical analyses.

Quantification and statistical analysis

Statistical analyses were performed in GraphPad Prism 10 (GraphPad Software). For comparisons between two independent groups, normality was evaluated using the Shapiro–Wilk test. When normality was not rejected ($p > 0.05$), equality of variance was assessed using an F test, and data were analyzed using a two-tailed unpaired *t* test when variances were comparable. For repeated-measures outcomes across times under two housing conditions, mixed-effects models (REML) were fitted with mouse identity as a random effect and with housing condition, time, and their interaction as fixed effects, followed by Bonferroni's multiple comparisons test. Model assumptions were evaluated by

inspection of residual plots, homoscedasticity plots, and Q–Q plots and did not reveal evidence of major assumption violations. Sample sizes (n), exact statistical tests, and the definition of error bars (mean ± SEM or median) are provided in the figure legend.

References

1. Fujishima K, Kiyonari H, Kurisu J, Hirano T, Kengaku M: **Targeted disruption of Sept3, a heteromeric assembly partner of Sept5 and Sept7 in axons, has no effect on developing CNS neurons.** *J Neurochem* 2007, **102:**77-92.
2. Ageta-Ishihara N, Fukazawa Y, Arima-Yoshida F, Okuno H, Ishii Y, Takao K, Konno K, Fujishima K, Ageta H, Hioki H, et al: **Septin 3 regulates memory and L-LTP-dependent extension of endoplasmic reticulum into spines.** *Cell Rep* 2025, **44:**115352.
3. Wagatsuma N, Terada Y, Okuno H, Ageta-Ishihara N: **Local connections among excitatory neurons underlie characteristics of enriched environment exposure-induced neuronal response modulation in layers 2/3 of the mouse V1.** *Front Syst Neurosci* 2025, **19:**1525717.
4. Ageta-Ishihara N, Fukumasu N, Fujii K, Koshidaka Y, Tanigaki K, Hiramoto T, Kang G, Hiroi N, Miyakawa T, Takao K, Kinoshita M: **Impairment of novelty-dependent hippocampal behavioural tagging in Septin5-deficient mice.** *Mol Brain* 2026, **19:**13.
5. Leger M, Quiedeville A, Bouet V, Haelewyn B, Boulouard M, Schumann-Bard P, Freret T: **Object recognition test in mice.** *Nat Protoc* 2013, **8:**2531-2537.
6. Ageta-Ishihara N, Fukumasu N, Sakakibara K, Fujii K, Koshidaka Y, Katsuragawa S, Tanigaki K, Hiramoto T, Kang G, Hiroi N, et al: **Septin5 deficiency impairs both recent and remote contextual fear memory.** *Mol Brain* 2025, **18:**85.
7. Parajuli LK, Ageta-Ishihara N, Ageta H, Fukazawa Y, Kinoshita M: **Methods for immunoblot detection and electron microscopic localization of septin subunits in mammalian nervous systems.** *Methods Cell Biol* 2016, **136:**285-294.

Supplementary Figure

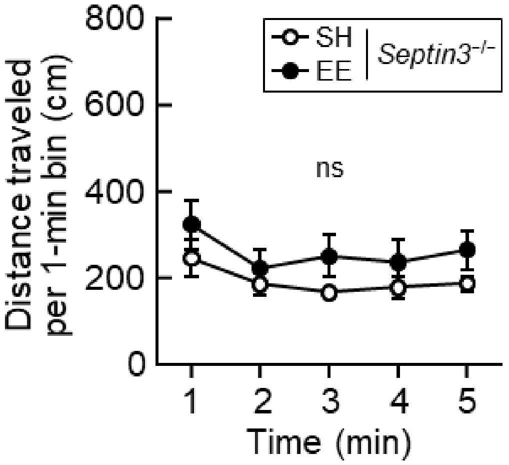

Figure S1

Minute-binned locomotor activity during the Day 6 novel object recognition test in *Septin3*$^{-/-}$ mice.

Locomotor activity during the novel object recognition test (Day 6; 5 min), expressed as distance traveled per 1-min bin. n = 7 male *Septin3*$^{-/-}$ mice per housing condition (SH and EE); mixed-effects model (REML) with time (min) as a within-subject repeated factor (subject = mouse) and housing condition as a fixed factor. No significant main effects of time ($F_{2.107, 25.28} = 2.49$, $p = 0.10$) or housing condition ($F_{1, 12} = 2.66$, $p = 0.13$), and no time × housing condition interaction ($F_{4, 48} = 0.20$, $p = 0.94$) were detected. Bonferroni's multiple comparisons test showed no significant differences at any time bin (all adjusted $p > 0.5$). Data are shown as mean ± SEM; ns, not significant.
